# Trend in survival after out-of-hospital cardiac arrest and its relationship with bystander cardiopulmonary resuscitation: a six-year prospective observational study in Beijing

**DOI:** 10.1186/s12872-021-02446-z

**Published:** 2021-12-31

**Authors:** Yuling Chen, Peng Yue, Ying Wu, Jia Li, Yanni Lei, Ding Gao, Jiang Liu, Pengda Han

**Affiliations:** 1 School of Nursing, Capital Medical University, No. 10, You An Men Wai Xi Tou Tiao, Fengtai District, Beijing, 100069 China; 2 Beijing Emergency Medical Center, No. 103, Qian Men Xi Da Jie, Xicheng District, Beijing, 100031 China

**Keywords:** Cardiac epidemiology, Critical care, Public health, Cardiac arrest, Cardiopulmonary resuscitation, Survival

## Abstract

**Background:**

Out-of-hospital cardiac arrest (OHCA), a global health problem with a survival rate ranging from 2 to 22% across different countries, has been a leading cause of premature death for decades. The aim of this study was to evaluate the trends of survival after OHCA over time and its relationship with bystander cardiopulmonary resuscitation (CPR), initial shockable rhythm, return of spontaneous circulation (ROSC), and survived event.

**Methods:**

In this prospective observational study, data of OHCA patients were collected following the “Utstein style” by the Beijing, China, Emergency Medical Service (EMS) from January 2011 (data from February to June in 2011 was not collected) to October 2016. Patients who had a cardiac arrest and for whom an ambulance was dispatched were included in this study. All cases were followed up to determine hospital discharge or death. The trend of OHCA survival was analyzed using the Chi-square test. The relationship among bystander CPR, initial shockable rhythm, ROSC, survived event, and OHCA survival rate was analyzed using multivariate path analyses with maximum standard likelihood estimation.

**Results:**

A total of 25,421 cases were transferred by the Beijing EMS; among them, 5042 (19.8%) were OHCA (median age: 78 years, interquartile range: 63–85, 60.1% male), and 484 (9.6%) received bystander CPR. The survival rate was 0.6%, which did not improve from 2012 to 2015 (*P* = 0.569). Overall, bystander CPR was indirectly associated with an 8.0% (*β* = 0.080, 95% confidence interval [*CI*] = 0.064–0.095, *P* = 0.002) increase in survival rate. The indirect effect of bystander CPR on survival rate through survived event was 6.6% (*β* = 0.066, 95% *CI* = 0.051–0.081, *P* = 0.002), which accounted for 82.5% (0.066 of 0.080) of the total indirect effect. With every 1 increase in survived event, the possibility of survival rate will directly increase by 53.5% (*β* = 0.535, 95% *CI* = 0.512–0.554, *P* = 0.003).

**Conclusions:**

The survival rate after OHCA was low in Beijing which has not improved between 2012 and 2015. The effect of bystander CPR on survival rate was mainly mediated by survived event.

*Trial registration*

Chinese Clinical Trial Registry: ChiCTR-TRC-12002149 (2 May, 2012, retrospectively registered). http://www.chictr.org.cn/showproj.aspx?proj=7400

**Supplementary Information:**

The online version contains supplementary material available at 10.1186/s12872-021-02446-z.

## Background

Out-of-hospital cardiac arrest (OHCA), a global health problem with a survival rate ranging from 2 to 22% across different countries [1], has been a leading cause of premature death from cardiovascular disease (CVD) for decades [2]. It is estimated that OHCA accounts for 50% of all CVD deaths in the world and for over 550,000 deaths in China annually [2]. The mortality rate from OHCA is projected to increase due to the rapid ageing of the population around the world [3] and in China [4].

Bystander cardiopulmonary resuscitation (CPR) has been proposed as the early link in the ‘Chain of Survival’ and is one of the most important modifiable factors in increasing the OHCA survival rates [5–7]. In the last decades, efforts have been made to increase the bystander CPR rate in most countries, especially in developed countries [8]. Along with the increased bystander CPR rate, OHCA survival rates have been increasing in some developed counties [9–11]. For instance, the bystander CPR rates and OHCA survival rates increased as follows: from 39.4% in 2006–2009 to 48.9% in 2014–2016, and from 10.4 to 14.9% in the same period of time in Canada [9]; from 28.2% in 2005 to 36.3% in 2012, and from 5.7 to 9.8% in the same period of time in the United States [11]; from 65.8% in 2006 to 81.2% in 2012, and from 16.2 to 19.7% in the same period of time in the Netherlands [10].

However, the survival rates in Asia–Pacific countries in which bystander CPR systems are also well developed, such as in Japan and Australia, are still unsatisfactory [12, 13]. In Japan, bystander CPR increased over time from 36.6% in 2005 to 48.4% in 2009, but the neurologically favorable survival rate only increased from 1.6% in 2005 to 2.8% in 2009 [12]. Similar results were observed in Victoria, Australia, where bystander CPR increased from 55% in 2011 to 60% in 2016, but no change was observed in survival rates in adults after OHCA in the same period of time [13].

Evidence has shown that the OHCA survival rate is closely associated with many pre-hospital modifiable factors besides bystander CPR [6, 9, 14, 15], including initial shockable rhythm, early defibrillation, return of spontaneous circulation (ROSC), and survived event (defined as ROSC sustained until the arrival at the emergency department and transfer of care to medical staff at the receiving hospital) [6, 9, 14, 15]. Most studies focused on exploring the relationship between prehospital factors and the survival rate after OHCA using multivariate logistic regression analysis [16–18]. The pathway among bystander CPR, other pre-hospital factors (such as initial shockable rhythm, ROSC, survived event), and survival rate after OHCA, however, remains unclear [19]. Previous prospective cohort studies found that bystander CPR may prolong the duration of the shockable rhythm [19], which might contribute to the increased ROSC rate [20] and survived event rate [21]. A cross-sectional observational study using the nationwide OHCA registry database in Korea [22] found that compared with no bystander CPR, bystander CPR was associated with increased rates of defibrillation and ROSC, therefore, increasing survival rate.

**Fig. 1:**
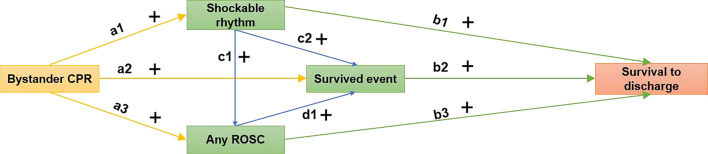
Proposed model of bystander cardiopulmonary resuscitation on survival of out-of-hospital cardiac arrest. ROSC, return of spontaneous circulation; CPR, cardiopulmonary resuscitation

Data from Canada, the United States, and the Netherlands, where OHCA survival rates have increased over time, have shown that most of the pre-hospital modifiable factors such as bystander CPR, initial shockable rhythm, ROSC, and survived event improved over time [9–11]. In Japan and Australia, although the rates of bystander CPR, initial shockable rhythm, and ROSC were increased, the rates of survival after OHCA and survived event were not improved over time [12, 13, 23]. Therefore, we hypothesized that the effect of bystander CPR on increasing OHCA survival rate was mainly mediated by survived event and partially mediated by shockable rhythm and ROSC (Fig. 1). The aims of this prospective observational study were to examine the pathway among bystander CPR, initial shockable rhythm, ROSC, survived event, and OHCA survival rate, and evaluated the temporal trend of survival after OHCA over time in Beijing, China.

## Methods

### Study design, setting and population

In this prospective observational study, data of OHCA patients were collected following the “Utstein style” [15] by the Beijing, China, Emergency Medical Service (EMS) from January 2011 (data from February to June in 2011 was not collected) to October 2016. Patients who had a cardiac arrest and for whom an ambulance was dispatched were included in this study. Cardiac arrest was defined as the absence of circulation signs, regardless of whether the assessment was made by the EMS or by a bystander [15]. All cases were followed up to determine hospital discharge or death. This study was approved by the Institutional Review Board of the Capital Medical University (No. 2010SY26). Informed consent from participants was waived by the Institutional Review Board of the Capital Medical University. The study was carried out in accordance with the Strengthening the Reporting of Observational studies in Epidemiology (STROBE) guidelines [24].

### System description

Beijing has a population of 21.73 million people, with approximately 18.80 million residing in urban areas across 16,410 km^2^ [25]. There are two separate EMS systems in Beijing: one is Beijing 120, a public medical rescue organization of the Beijing Municipal Health Commission; the other is Beijing 999, which is subordinate to the Beijing Red Cross. Although people call 120 or 999 randomly, the reported number of dispatches for patients who called 120 was three times higher than dispatches for patients who called 999. Only cases of cardiac arrest that occurred in the Beijing urban area and that were served by Beijing 120 were included in this study (Fig. S1, Additional file 1).

Rescue ambulances equipped with electrocardiograph machines and defibrillators were dispatched to transfer OHCA patients to hospitals in this study. All ambulances were staffed with one physician, one nurse, and one driver, and they received special training in performing CPR and advanced cardiac life support (ACLS). The EMS physicians and nurses performed CPR and ACLS when indicated (except hypothermia therapy) either on site or on the ambulance following the resuscitation guidelines [26–28]. The patients were assessed and treated by physicians in the emergency department after transfer to the receiving hospitals prior to hospital admission. It should be noted that automated external defibrillators (AEDs) were not yet available in public areas during the study period except in the Capital International Airport Terminal Three.

### Data collection procedures

Patients’ characteristics were collected prospectively on the ambulances by EMS physicians who were trained to use an Utstein registration sheet for documenting OHCA events. For each OHCA case, the EMS physician completed a registration sheet including the following: (1) general demographic information, such as age and gender; (2) first witness and initial management measures, such as the location of the cardiac arrest, whether the patients received bystander CPR, and whether the witness was trained in CPR; (3) assessment and treatment by the EMS crew, such as patients’ mental status, vital signs, type of initial rhythm when the ambulance arrived, type of rhythm when the patient arrived at the emergency department, defibrillation, tracheal intubation, medication administration, probable cause of the cardiac arrest (cardiac, respiratory, traumatic, drowning, asphyxia, suicide, drug overdose, others), cardiovascular history, achieved ROSC, and survived event; and (4) timing-related information, such as the date and time of the cardiac arrest, time of the emergency call, and time the ambulance arrived. In addition, information was collected by reviewing the medical records regarding whether the patient died in the hospital, survived to admission, and survived to discharge.

### Operational definition

Any ROSC was defined “according to a clinical assessment that comprised a palpable pulse or a blood pressure at any point during the resuscitation attempt” [15]. A survived event was defined as “ROSC sustained until the arrival at the emergency department and transfer of care to medical staff at the receiving hospital” [15]. Survival to admission was defined as “patients having ROSC after the treatment in the emergency department and being hospitalized” [15]. Survival to discharge was defined as patients having ROSC at the point of hospital discharge. A resuscitation attempted by EMS personnel was defined as a resuscitation when EMS personnel perform chest compressions or attempt defibrillation [15].

### Data analysis

First, we analyzed the characteristics and survival rates among five groups of patients: (1) all OHCA patients, (2) those that EMS attempted to resuscitate, (3) those that EMS did not attempt to resuscitate, (4) those patients who were transported to a hospital with ROSC (that is, those who survived the event), and (5) those patients who were transported to a hospital with ongoing bystander CPR.

Second, we compared the differences in characteristics between patients who survived to discharge and those who did not survive to discharge, among all OHCA patients after excluding 28 patients who were lost-to-follow-up at hospital discharge. Student *t* or Mann–Whitney *U* tests were used for continuous data. Chi-square or Fisher’s exact tests were carried out for categorical data. We performed the same analyses among those that EMS attempted to resuscitate, those who survived the event, and those who were transported to a hospital with ongoing bystander CPR.

Third, to evaluate the trends of survival rates and pre-hospital factors over time, a trend chi-square test (linear-by-linear) was conducted for dichotomous data; linear regression was used in case of normally distributed continuous variables. The Jonckheere-Terpstra test was used for non-normally distributed continuous variables. Given that OHCA cases from February to June in 2011 and from November to December in 2016 were not collected, we analyzed only those trends from 2012 to 2015. Analyses were conducted using IBM SPSS Statistics software (version 24, IBM Corp., Armonk, New York, USA).

Finally, to analyze the relationship among bystander CPR, initial shockable rhythm, any ROSC, survived event, and OHCA survival rate, multivariate path analyses with maximum standard likelihood estimation were conducted using IBM SPSS Amos 24.0. The multivariate path analyses were performed among all OHCA patients (model 1) and among those that EMS attempted to resuscitate (model 2). Three steps were carried out to test the mediation effect as follows: (1) The joint significance of the mediation path was used to indicate that full mediation had occurred. (2) The product of the path was calculated to assess the amount of mediation effect. (3) A bootstrapping method with bias-corrected confidence estimates was used to assess the significance of the indirect path.

The acceptable model fitting values for these measures were defined as follows: (1) nonsignificant chi‐square value; (2) comparative fit index > 0.90; (3) goodness of fit index > 0.90; (4) Tucker Lewis index > 0.90; (5) root mean square error of approximation < 0.08; and (6) standardized root mean squared residual < 0.08 [29]. Effect sizes were measured using the standardized estimates and were evaluated at the following levels: 0.1 = small; 0.3 = medium; 0.5 = large. The model was modified until its indices fit well.

A series of sensitivity analyses were conducted to enhance the rigor of the study findings. First, we performed a path model (model 3) assuming that survival to admission was a mediator between survived event and survival to discharge. Second, we conducted a path model (model 4) using survival to admission as a dependent variable. Third, we performed three path models (models 5, 6, and 7) assuming that all patients who were lost-to-follow-up were dead.

## Results

**Fig. 2:**
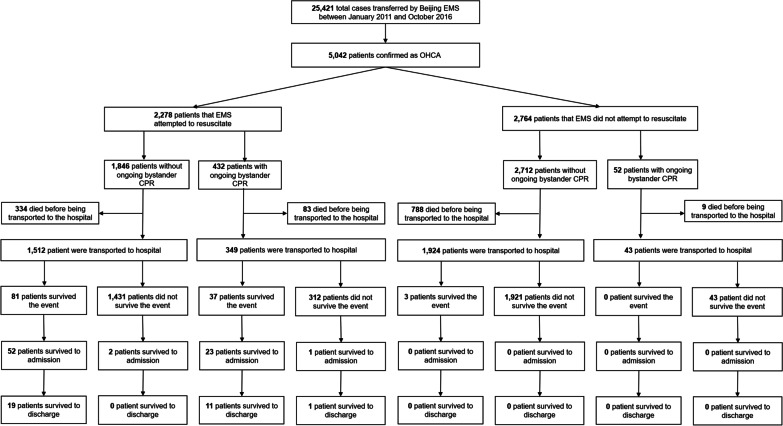
Flow chat of the study. OHCA, Out-of-Hospital Cardiac Arrest; EMS, Emergency Medical Services; ROSC, return of spontaneous circulation

Figure 2 displays the flowchart of sample recruitment and the number of patients in different stages of resuscitation. A total of 25,421 case were transferred by Beijing EMS during the study period; among them, 5042 patients were confirmed as OHCA and were included in the final analysis. Characteristics of OHCA in Beijing during the study period are shown in Table 1. Among 5,042 patients confirmed as OHCA, 3,032 (60.1%) were male, with a median age of 78 (interquartile range [IQR]: 63–85) years, and 88.3% (4,452 cases) were of cardiac etiology. Of all the enrolled OHCA cases, 484 (9.6%) received bystander CPR before the arrival of EMS personnel; 2,278 (45.2%) were recorded as resuscitation attempted by EMS personnel; 2,764 (54.8%) patients did not receive resuscitation because the victims were obviously dead.

**Table 1:** Characteristics of out-of-hospital cardiac arrest in Beijing

Characteristics	Overall (n = 5042)	EMS attempted to resuscitate (N = 2278)	EMS did not attempt to resuscitate (N = 2764)	Transported to hospital with ROSC (N = 121)	Transported to hospital with ongoing bystander CPR (N = 392)
Age, median (IQR)	78 (63–85)	70 (57–80)	82 (73–87)	69 (55–79)	68 (57–80)
Gender					
Male	3032 (60.1)	1574 (69.1)	1458 (52.7)	67 (55.4)	282 (71.9)
Female	2010 (39.9)	704 (30.9)	1306 (47.3)	54 (44.6)	110 (28.1)
Arrest location					
At home	4319 (85.7)	1727 (75.8)	2592 (93.8)	83 (68.6)	299 (76.3)
At public place	723 (14.3)	551 (24.2)	172 (6.2)	38 (31.4)	93 (23.7)
Witnessed status					
Non-witnessed	2837 (56.3)	854 (37.5)	1983 (71.8)	26 (21.5)	122 (31.1)
Witnessed	2203 (43.7)	1424 (62.5)	779 (28.2)	95 (78.5)	270 (68.9)
Bystander CPR					
Yes	484 (9.6)	432 (19.0)	52 (1.9)	37 (30.6)	392 (100.0)
No	4558 (90.4)	1846 (81.0)	2712 (98.1)	84 (69.4)	0
Bystanders trained in CPR					
Yes	469 (9.3)	319 (14.0)	150 (5.4)	37 (30.6)	223 (56.9)
No	4573 (90.7)	1959 (86.0)	2614 (94.6)	84 (69.4)	169 (43.1)
First monitored shockable rhythm					
Yes	162 (3.2)	149 (6.5)	13 (0.5)	26 (21.5)	50 (12.8)
No	4880 (96.8)	2129 (93.5)	2751 (99.5)	95 (78.5)	342 (87.2)
Etiology					
Cardiac	4452 (88.3)	2045 (89.8)	2407 (87.1)	104 (86.0)	358 (91.3)
Non-cardiac	590 (11.7)	233 (10.2)	357 (12.9)	17 (14.0)	34 (8.7)
Previous CVD					
Yes	3249 (64.8)	1436 (63.0)	1929 (66.1)	58 (47.9)	261 (66.6)
No	1765 (35.2)	842 (37.0)	936 (33.9)	63 (52.1)	131 (33.4)
Response time (min), median (IQR)	15 (12–20)	15 (11–19)	16 (12–20)	14 (11–18)	15 (12–19)
Defibrillated by EMS	359 (7.1)	359 (15.8)	0	42 (35.6)	82 (20.9)
Any ROSC					
Yes	95 (1.9)	86 (3.8)	9 (0.3)	19 (15.0)	25 (6.4)
No	4947 (98.1)	2191 (96.2)	2754 (99.7)	102 (85.0)	367 (93.6)
Survived event					
Yes	121 (2.4)	118 (5.2)	3 (0.1)	121 (100.0)	37 (9.4)
No	4921 (97.6)	2160 (94.8)	2761 (99.9)	0	355 (90.6)
Survived to admission					
Yes	78 (1.5)	78 (3.4)	0	75 (62.0)	24 (6.1)
No	4964 (98.5)	2200 (96.6)	2764 (100.0)	46 (38.0)	368 (93.9)
Survived to discharge					
Yes	31 (0.6)	31 (1.4)	0	30 (24.8)	12 (3.1)
No	4983 (98.8)	2219 (97.4)	2764 (100.0)	65 (53.7)	377 (96.2)
Lost to follow-up	28 (0.6)	28 (1.2)	0	26 (21.5)	3 (0.8)

Data are shown as frequency (percentage) or median (interquartile range). CPR, cardiopulmonary resuscitation; CVD, cardiovascular disease; IQR, interquartile range; ROSC, return of spontaneous circulation

### Survival rate

As shown in Table 1, overall, the rates of patients who survived to discharge, survived to admission, and survived the event were 0.6% (31 of 5014, excluding 28 patients lost-to-follow-up at hospital discharge), 1.5% (78 of 5042), and 2.4% (121 of 5042), respectively. Among patients that EMS attempted to resuscitate, the rates of patients who survived to discharge, survived to admission, and survived the event were 1.4%, 3.4%, and 5.2%, respectively. Among patients that EMS did not attempt to resuscitate, only 3 patients survived event, and no case survived to admission or discharge. Among patients who were transported to hospital with ongoing bystander CPR, the rates of patients who survived to discharge, survived to admission, and survived the event were 3.1%, 6.1%, and 9.4%, respectively. As shown in Fig. 2, among patients who survived to admission, 39.7% (31 of 78) survived to discharge, 24.4% (19 of 78) died after being hospitalized, and 35.9% (28 of 78) were lost-to-follow-up.

### Trends in survival rates after OHCA from 2012 to 2015

Table 2 displays the trends in survival rates after OHCA, demographic characteristics of enrolled patients (age and gender), bystander CPR, bystander CPR training, and response time from 2012 to 2015. No changes were observed in the rates of survival to discharge (*P*_for trend_ = 0.569, excluding 28 patients lost-to-follow-up at hospital discharge), survival to admission (*P*_for trend_ = 0.732), or survived event (*P*_for trend_ = 0.659) from 2012 to 2015 for all OHCA patients. For patients that EMS attempted to resuscitate, the rates were also stable for survival to discharge (*P*_for trend_ = 0.846, excluding 28 patients who lost to follow-up at hospital discharge), survival to admission (*P*_for trend_ = 0.709), or survived event (*P*_for trend_ = 0.749) from 2012 to 2015. A small increase in the median age was found, from 77 (IQR: 62–84) years in 2012 to 79 (IQR: 64–85) years in 2015 (*P*_for trend_ = 0.004), but no change was found for gender (*P*_for trend_ = 0.815). Although no improvement was observed in bystander CPR training from 2012 to 2015 (*P*_for trend_ = 0.133), the rate of bystander CPR increased from 7.1% to 11.2% (*P*_for trend_ = 0.005) during this period, with a relative increase rate of 57.7%. A small increase in the response time was detected from 15 (IQR: 11–19) minutes to 16 (IQR: 12–20) minutes during this period (*P*_for trend_ < 0.001).

**Table 2:** Trends in the survival after OHCA, pre-hospital factors from 2012 to 2015

Variables	2012	2013	2014	2015	*P*_for trend_
OHCA patients, n	920	1021	625	1174	
Age (years, median, IQR)	77 (62–84)	77 (63–84)	77 (62–85)	79 (64–85)	0.004
Male (n, %)	547 (59.5)	707 (62.5)	406 (58.4)	791 (60.2)	0.815
Response time (minutes, median, IQR,)	15 (11–19)	15 (12–20)	16 (12–20)	16 (12–20)	< 0.001
Bystander CPR (n, %)	65 (7.1)	114 (10.1)	59 (8.5)	147 (11.2)	0.005
Bystanders trained in CPR (n, %)	79 (8.6)	111 (9.8)	70 (10.1)	139 (10.6)	0.133
Survived event (EMS attempted resuscitation, n, %)	20 (4.4)	28 (5.0)	16 (5.2)	25 (4.8)	0.749
Survived event (all OHCA patients, n, %)	20 (2.2)	28 (2.5)	17 (2.4)	26 (2.0)	0.659
Survival to admission (EMS attempted resuscitation, n, %)	12 (2.6)	21 (3.7)	10 (3.3)	17 (3.3)	0.709
Survival to admission (all OHCA patients, n, %)	12 (1.3)	21 (1.9)	11 (1.6)	17 (1.3)	0.732
Survived to discharge (EMS attempted resuscitation, n, %)	5 (1.1)	8 (1.4)	5 (1.6)	5 (1.0)	0.846
Survived to discharge (all OHCA patients, n, %)	5 (0.5)	8 (0.7)	6 (0.9)	5 (0.4)	0.569

CPR, cardiopulmonary resuscitation; EMS, Emergency Medical Services; OHCA, Out-of-Hospital Cardiac Arrest; IQR, interquartile range

### Bystander CPR and bystander CPR training

As shown in Table 1, among all enrolled OHCA cases, 484 (9.6%) received bystander CPR (271 of 484 of these bystanders had CPR training). The differences in characteristics between patients who survived to discharge and those who did not survive to discharge were summarized in Table 3 and Table S1 (Additional file 1). As shown in Table 3, although significant differences in the survival rates were observed among victims who received or did not receive bystander CPR, the rates of survival to discharge were both very low: only 2.5% (12 of 481) and 0.4% (19 of 4533) among all OHCA patients (*P* < 0.001); and 2.8% (12 of 429) and 1.0% (19 of 1821) among those that EMS attempted to resuscitate (*P* < 0.001). As shown in Table S1 (Additional file 1), among patients who survived the event, no difference was found in the rate of survival after OHCA for victims who received or did not receive bystander CPR (*P* = 0.904). Bystanders with previous CPR training did not change the rate of survival to discharge whether they performed CPR or not (Figure S2, Additional file 1). There was no dispatcher-assisted bystander CPR or bystander AED use during the study period.

**Table 3:** Comparisons in characteristics between patients who survived to discharge and those who did not

Characteristics	All patients (N = 5014) ^&^			EMS attempted resuscitation (N = 2250) ^&^		
	Survival to Discharge (n = 31)	Non-survival (n = 4983)	*P*	Survival to Discharge (n = 31)	Non-survival (n = 2219)	*P*
Age, median (IQR)	63 (52–73)	78 (63–85)	< 0.001	63 (52–73)	70 (57–80)	0.021
Gender			0.898			0.331
Male	19 (61.3)	2998 (60.2)		19 (61.3)	1540 (69.4)	
Female	12 (38.7)	1985 (39.8)		12 (38.7)	679 (30.6)	
Arrest location			< 0.001			0.018
At home	18 (58.1)	4286 (86.0)		18 (58.1)	1694 (76.3)	
At public place	13 (41.9)	697 (14.0)		13 (41.9)	525 (23.7)	
Witnessed status			< 0.001			0.079
Non-witnessed	7 (22.6)	2826 (56.7)		7 (22.6)	843 (38.0)	
Witnessed	24 (77.4)	2155 (43.3))		24 (77.4)	1376 (62.0)	
Bystander CPR			< 0.001			0.005
Yes	12 (38.7)	469 (9.4)		12 (38.7)	417 (18.8)	
No	19 (61.3)	4514 (90.6)		19 (61.3)	1802 (81.2)	
Bystanders trained in CPR			< 0.001			< 0.001
Yes	12 (38.7)	450 (9.0)		12 (38.7)	300 (13.5)	
No	19 (61.3)	4533 (91.0)		19 (61.3)	1919 (86.5)	
First monitored shockable rhythm			< 0.001			< 0.001
Yes	11 (35.5)	145 (2.9)		11 (35.5)	132 (5.9)	
No	20 (64.5)	4838 (97.1)		20 (64.5)	2087 (94.1)	
Etiology			0.445			0.279
Cardiac	26 (83.9)	4400 (88.3)		26 (83.9)	1993 (89.8)	
Non-cardiac	5 (16.1)	583 (11.7)		5 (16.1)	226 (10.2)	
Previous CVD			0.022			0.036
Yes	14 (45.2)	3235 (64.9)		14 (45.2)	1407 (63.4)	
No	17 (54.8)	1748 (35.1)		17 (54.8)	812 (36.6)	
Response time (min), median (IQR)	14 (10–19)	15 (12–20)	0.286	14 (10–19)	15 (11–19)	0.619
Any ROSC			< 0.001			< 0.001
Yes	6 (19.4)	84 (1.7)		6 (19.4)	75 (3.4)	
No	25 (80.6)	4898 (98.3)		25 (80.6)	2144 (96.6)	
Survived event			< 0.001			< 0.001
Yes	30 (96.8)	65 (1.3)		30 (96.8)	62 (2.8)	
No	1 (3.2)	4918 (98.7)		1 (3.2)	2157 (97.2)	
Survived to admission			< 0.001			< 0.001
Yes	31 (100.0)	19 (0.4)		31 (100.0)	19 (0.9)	
No	0	4964 (99.6)		0	2200 (99.1)	

Data are shown as frequency (percentage) or median (interquartile range). CPR, cardiopulmonary resuscitation; CVD, cardiovascular disease; IQR, interquartile range; ROSC, return of spontaneous circulation. ^&^ Excluding 28 patients who were lost to follow-up

### Initial rhythm

Among the enrolled OHCA cases, only 162 (3.2%) had initial shockable rhythm (including ventricular fibrillation or pulseless ventricular tachycardia). Cases with initial non-shockable rhythm (which accounted for 93.0% [4689]) were pulseless, with no electrical rhythm and were in asystole. There were 191 cases (3.8%) with other initial rhythm noted as myocardial infarction, atrial fibrillation, or ventricular escape; however, cardiac arrest occurred during the transfer to the hospital among those cases. Among the 191 cases, only 1 (0.5%) victim survived to discharge. As shown in Table 3, among all OHCA patients, the rate of survival to discharge was 7.1% (11 of 156) in those OHCA victims with initial shockable rhythm, and 0.4% (20 of 4858) in those with initial non-shockable rhythm (including other initial rhythm; *P* < 0.001). Among those that EMS attempted to resuscitate, the rate of survival to discharge was 7.7% (11 of 143) in those OHCA victims with initial shockable rhythm, and 0.9% (20 of 2,107) in those with initial non-shockable rhythm (including other initial rhythm; *P* < 0.001; Table 3).

### Any return of spontaneous circulation

Any ROSC was achieved in 95 OHCA cases (1.9%). Among patients who had any ROSC, 18.9% (18 of 95) survived the event, 14.7% (14 of 95) survived to admission, and 6.3% (6 of 95) survived to discharge. As shown in Table 3, among all OHCA patients, the rate of survival to discharge was 6.7% (6 of 90) in those OHCA victims with any ROSC and 0.5% (25 of 4923) in those without (*P* < 0.001). Among those that EMS attempted to resuscitate, the rate of survival to discharge was 7.4% (6 of 81) in those OHCA victims with any ROSC and 1.2% (25 of 2,169) in those without ROSC (*P* < 0.001).

### The relationship and pathways among bystander CPR, other prehospital factors, and the survival rate

**Fig. 3:**
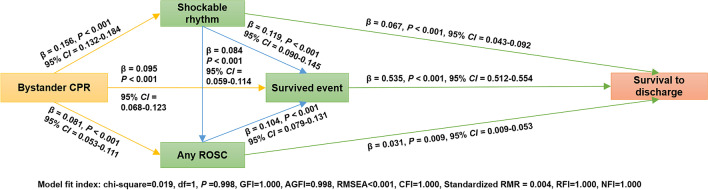
Multivariate path analysis model 1 (among all OHCA patients). N = 5014, excluding 28 patients who lost to follow-up. OHCA, Out-of-Hospital Cardiac Arrest; ROSC, return of spontaneous circulation; CPR, cardiopulmonary resuscitation; GFI, goodness-of-fit index; AGFI, adjusted goodness-of-fit index; RMSEA, root mean square error of approximation; CFI, comparative fit index; Standardized RMR, standardized root mean squared residual; RFI, relative fit index; NFI, normed fit index; CI, confidence interval; β, standardized estimate

The result of the multivariate path analysis among all OHCA patients is displayed in Fig. 3 (model 1) and Table 4. The tested model had a proper fit. In this model, we excluded 28 patients lost-to-follow-up. As shown in Table 4, no direct relationship was found in bystander CPR and OHCA survival. The indirect effect of bystander CPR on OHCA survival was significant and was mainly mediated by a survived event. Overall, bystander CPR was indirectly associated with an 8.0% (*β* = 0.080, 95% confidence interval [*CI*] = 0.064–0.095, *P* = 0.002) increase in the possibility of survival rate (Table 4, total indirect effect). The total indirect effect of bystander CPR on the survival rate through a survived event was 6.6% (*β* = 0.066, 95% *CI* = 0.051–0.081, *P* = 0.002; Table 4, indirect effect), which accounted for 82.5% (0.066 of 0.080) of the total indirect effect.

**Table 4:** The direct effect, indirect effect, and total effect of bystander CPR on the survival after OHCA among all patients (N = 5014) ^&^

	Unstandardized Estimate	Standardized Estimate	Bootstrap Bias-corrected 95% confidence interval		
			Lower	Upper	*P*-value
Multivariate Path Analyses Model of Bystander CPR on OHCA Survival					
Bystander CPR-Initial Shockable Rhythm	0.092	0.156	0.132	0.184	0.001
Bystander CPR-Survived Event	0.044	0.095	0.068	0.123	0.002
Bystander CPR-ROSC	0.036	0.081	0.053	0.111	0.002
Initial Shockable Rhythm-ROSC	0.064	0.084	0.059	0.114	0.001
Initial Shockable Rhythm-Survived Event	0.093	0.119	0.09	0.145	0.002
ROSC-Survival Event	0.107	0.104	0.079	0.131	0.002
Initial Shockable Rhythm-Survival to Discharge	0.030	0.067	0.043	0.092	0.002
Survived Event-Survival to Discharge	0.308	0.535	0.512	0.554	0.003
ROSC-Survival to Discharge	0.018	0.031	0.009	0.053	0.007
Effect of Bystander CPR on OHCA Survival					
Total effect	0.021	0.080	0.064	0.095	0.002
Total direct effect	0.000	0.000	0.000	0.000	0.000
Total indirect effect	0.021	0.080	0.064	0.095	0.002
Mediation Analyses and Potential Paths					
P_1_: Bystander CPR-Initial Shockable Rhythm-Survival to Discharge	0.003	0.010	0.007	0.015	0.002
P_2_: Bystander CPR-Survived Event-Survival to Discharge	0.014	0.051	0.036	0.066	0.002
P_3_: Bystander CPR-ROSC-Survival to Discharge	0.001	0.003	0.001	0.005	0.006
P_4_: Bystander CPR-Initial Shockable Rhythm-ROSC-Survival to Discharge	0.000	0.000	0.000	0.001	0.004
P_5_: Bystander CPR-Initial Shockable Rhythm-ROSC-Survived Event-Survival to Discharge	0.000	0.001	0.000	0.001	0.001
P_6_: Bystander CPR-Initial Shockable Rhythm-Survived Event-Survival to Discharge	0.003	0.010	0.007	0.013	0.001
P_7_: Bystander CPR-ROSC-Survived Event-Survival to Discharge	0.001	0.004	0.003	0.007	0.002
The Total Indirect Effect of Bystander CPR on OHCA Survival through Survived Event					
Mediator: Survived Event (P_2_ + P_5_ + P_6_ + P_7_)	0.018	0.066	0.051	0.081	0.002

Bystander CPR: bystander cardiopulmonary resuscitation. ROSC: return of spontaneous circulation. OHCA, out-of-hospital cardiac arrest. P: path. ^&^ Excluding 28 patients who lost to follow-up

A survived event has a significant impact on survival after OHCA. With every 1% increase in the survived event, the possibility of the survival rate will directly increase by 53.5% (*β* = 0.535, 95% *CI* = 0.512–0.554, *P* = 0.003; Table 4). As shown in Fig. 3, although initial shockable rhythm and any ROSC were also positively related to the survival rate, they directly increased the chance of survival by only 6.7% and 3.1%, respectively; their indirect effect on the survival rate through a survived event, however, was 6.4% and 5.6%, respectively. Both the effect of initial shockable rhythm and any ROSC on the survival rate were mediated by a survived event, with every 1% increase in the rates of initial shockable rhythm and any ROSC directly leading to an 11.9% and 10.4% increase in the possibility of a survived event, respectively (Fig. 3).

When analyzing the relationships between bystander CPR, initial shockable rhythm, any ROSC, survived event, and survival rate, we found that bystander CPR affected the survival rate mainly through the “*Bystander CPR-Survived Event-Survival to Discharge*” path (P2, Table 4). The total indirect effect of bystander CPR on the survival rate through this path was 5.1% (*β* = 0.051, 95% *CI* = 0.036–0.066, *P* = 0.002).

**Fig. 4:**
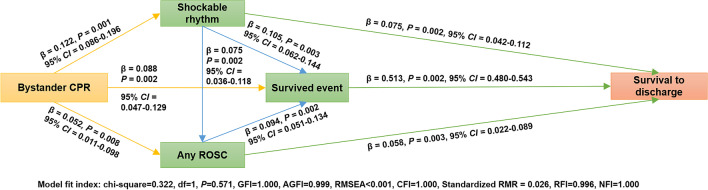
Multivariate path analysis model 2 (among patients that EMS attempted to resuscitate). N = 2250, excluding 28 patients lost-to-follow-up. EMS, Emergency Medical Services; OHCA, Out-of-Hospital Cardiac Arrest; ROSC, return of spontaneous circulation; CPR, cardiopulmonary resuscitation; GFI, goodness-of-fit index; AGFI, adjusted goodness-of-fit index; RMSEA, root mean square error of approximation; CFI, comparative fit index; Standardized RMR, standardized root mean squared residual; RFI, relative fit index; NFI, normed fit index; CI, confidence interval; β, standardized estimate

The result of the multivariate path analysis among those patients that EMS attempted to resuscitate is displayed in Fig. 4 (model 2) and Table 5. This tested model also had a proper fit. The pathways and relationships among bystander CPR, initial shockable rhythm, any ROSC, survived event, and survival rate in those patients that EMS attempted to resuscitate were consistent with the results in all OHCA patients.

**Table 5:** The direct effect, indirect effect, and total effect of bystander CPR on the survival after OHCA among patients that EMS attempted to resuscitate (N = 2250)^&^

	Unstandardized Estimate	Standardized Estimate	Bootstrap Bias-corrected 95% confidence interval		
			Lower	Upper	*P*-value
Multivariate Path Analyses Model of Bystander CPR on OHCA Survival					
Bystander CPR-Initial Shockable Rhythm	0.076	0.122	0.086	0.196	0.001
Bystander CPR-Survived Event	0.045	0.088	0.047	0.129	0.002
Bystander CPR-ROSC	0.024	0.052	0.011	0.098	0.008
Initial Shockable Rhythm-ROSC	0.055	0.075	0.036	0.118	0.002
Initial Shockable Rhythm-Survived Event	0.085	0.105	0.062	0.144	0.003
ROSC-Survival Event	0.103	0.094	0.051	0.134	0.002
Initial Shockable Rhythm-Survival to Discharge	0.034	0.075	0.042	0.112	0.002
Survived Event-Survival to Discharge	0.290	0.513	0.480	0.543	0.002
ROSC-Survival to Discharge	0.036	0.058	0.022	0.089	0.003
Effect of Bystander CPR on OHCA Survival					
Total effect	0.019	0.068	0.045	0.089	0.002
Total direct effect	0.000	0.000	0.000	0.000	0.000
Total indirect effect	0.019	0.068	0.045	0.089	0.002
Mediation Analyses and Potential Paths					
P_1_: Bystander CPR-Initial Shockable Rhythm-Survival to Discharge	0.003	0.009	0.005	0.016	0.001
P_2_: Bystander CPR-Survived Event-Survival to Discharge	0.013	0.045	0.024	0.066	0.002
P_3_: Bystander CPR-ROSC-Survival to Discharge	0.001	0.003	0.001	0.007	0.004
P_4_: Bystander CPR-Initial Shockable Rhythm-ROSC-Survival to Discharge	0.000	0.001	0.000	0.001	0.001
P_5_: Bystander CPR-Initial Shockable Rhythm-ROSC-Survived Event-Survival to Discharge	0.000	0.000	0.000	0.001	0.001
P_6_: Bystander CPR-Initial Shockable Rhythm-Survived Event-Survival to Discharge	0.002	0.007	0.004	0.011	0.001
P_7_: Bystander CPR-ROSC-Survived Event-Survival to Discharge	0.001	0.003	0.001	0.006	0.005
The Total Indirect Effect of Bystander CPR on OHCA Survival through Survived Event					
Mediator: Survived Event (P_2_ + P_5_ + P_6_ + P_7_)	0.016	0.055	0.033	0.075	0.002

Bystander CPR: bystander cardiopulmonary resuscitation. ROSC: return of spontaneous circulation. OHCA, out-of-hospital cardiac arrest. P: path. ^&^ Excluding 28 patients lost-to-follow-up

The sensitivity analyses are displayed in the Figs. S3–S7 (models 3–7). The sensitivity analyses showed that the indirect effect of bystander CPR on survival rate through increasing the survived event rate remained unchanged.

## Discussions

To the best of our knowledge, this study was the first large prospective study that examined the trend of OHCA survival rate, survived event, and bystander CPR in China over the study period, and the possible causal relationship between bystander CPR, other pre-hospital factors and survival rates after OHCA. We found that the effect of bystander CPR on increasing the OHCA survival rate was mainly mediated by a survived event. Although the rate of bystander CPR increased from 2012 to 2015 in Beijing, the survival rate after OHCA was extremely low and had not improved over the same period possibly due to the fact that the rate of survived event has not increased during this period.

Our findings confirm the hypothesis that the effect of bystander CPR on increasing OHCA survival rate is mainly mediated by increased survived event rate; only increasing the rates of shockable rhythm or any ROSC without increasing survived event rate had very limited effect on the survival rates for patients with OHCA. However, given that shockable rhythm and ROSC were positively associated with the survival rate after OHCA [9, 14, 19, 20], most studies did not focus on survived event rate, but rather focused on whether increasing bystander CPR could increase the likelihood of shockable rhythm and ROSC [20, 30].

Takahashi and colleagues [30] found that an increased bystander CPR rate was significantly associated with an increased shockable rhythm rate, ROSC rate, and improved survival rates. Takahashi’s findings support the findings in developed countries [9–11], but they do not explain the results in Japan and Australia [12, 13], where the survived event rate did not improve over time and the survival rate did not increase either [13], or only increased by 0.8% [12]. In our study, the results of multivariate path analysis suggested that whether bystander CPR can increase the OHCA survival rate depends largely on the presence of a survived event, a term which has been mentioned in the Utstein Resuscitation Registry Templates for OHCA since 2004 [15, 31], but has rarely been reported as a predicting factor of OHCA survival rate in most of the current published studies [9, 14, 32].

It is well known in general that patients who had initial shockable rhythm—no matter with or without bystander CPR—had higher proportions of favorable outcomes [23, 28]. The path analysis of our study suggests that initial shockable rhythm was positively associated with increased survival rate, which was consistent with previous studies [23, 28]. Moreover, our study highlights the role of survived event in increasing the survival rate after OHCA. Our study found that for patients with initial shockable rhythm, the chance of survival increased by only 6.7% if the patients had not survive the event; the possibility of a survival rate could increase to 11.9% if the patients survived the event. In line with previous reports [6, 22], we also indicated that ROSC was associated with an improved survival rate. Of note, our study suggested that despite patients having achieved any ROSC after defibrillation, the possibility of survival rate increased by only 3.1% if the patient did not survive the event; however, the possibility of a survival rate could increase to 10.4% if the patients survived the event.

The reasons for our findings are obvious. Cardiac arrest is a disorder characterized by cessation of the pumping function of the heart, which usually causes death if the heart does not achieve ROSC within minutes and sustain ROSC at the emergency department (that is, survived event) [33]. The sinoatrial node is the primary cardiac pacemaker with the highest automaticity of heart [34]. The sinoatrial node cells may suffer from cardiac ischemia when patients do not survive the event after OHCA [35]. The cardiac ischemia may lead to a reduced automaticity in the sinoatrial node cells, and result in decreased energy supply for supporting the contractility of the cardiac myocytes [35]. The survived event after OHCA depends largely on whether the sinoatrial node cells return automaticity and contractility within the timeframe of cardiac ischemia [36, 37]. One study found that bystander CPR may help the cardiac myocytes to recover automaticity and contractility, and improve the possibility of the rate of survived event [38]. Thus, after receiving bystander CPR or defibrillation, if the patient could achieve ROSC quickly and survived the event, the sinoatrial node cells could return automaticity and contractility in time, and thereby the chance of survival for patients with OHCA could increase [37]. To increase the survival rate after OHCA in China, more focus on the quality of bystander CPR and the chance of a survived event are urgently needed [39].

### Limitations

This study has several limitations. First, as only 31 patients survived to discharge, this study may not have the power to explore the effect of the study factors on survival rates. The overall survival rates, however, were still very low (1.2%) even if those patients who were lost-to-follow-up were considered as survivals to discharge. To further verify our findings and to explore the effect of each factor on survival rates, a large-scale study is needed that includes more patients who survived to discharge. Second, this study was not a nationwide OHCA registry prospective study, which may hinder the generalizability of our findings. However, controlling for the inclusion bias, we consecutively screened OHCA patients from the official EMS in Beijing which has the largest number of emergency patients transferred. Third, AED was not available in public areas in Beijing; therefore, bystander defibrillation was not provided during the study period, which might reduce the effect of initial shockable rhythm on OHCA survival. Moreover, in this study, we can not test the effect of early defibrillation on increasing the survival rate. More studies exploring the pathways among early defibrillation, bystander CPR, other predictors of OHCA survival (ROSC, initial shockable rhythm, survived event), and survival rate are warranted. Finally, we did not collect data from February to June in 2011 and from November to December in 2016, which might bias the results of the study.

## Conclusions

The rate of survival after OHCA was still extremely low in Beijing, and did not improve from 2012 to 2015. The effect of bystander CPR in increasing survival rates of OHCA was mainly mediated by a survived event. This suggests that efforts, such as facilitating high-quality CPR training to increase the rate of survived event, will have a substantial effect on improving the survival rate after OHCA in Beijing.

## Supplementary Information

**Additional file 1. Supplemental Figure 1**. The map of Emergency Medical Services centers in Beijing.** Supplemental Figure 2**. Survival rates of out-of-hospital cardiac arrest among bystanders with previous CPR training.** Supplemental Figure 3**. Sensitivity analyses: multivariate path analysis model 3.** Supplemental Figure 4**. Sensitivity analyses: multivariate path analysis model 4.** Supplemental Figure 5**. Sensitivity analyses: multivariate path analysis model 5.** Supplemental Figure 6**. Sensitivity analyses: multivariate path analysis model 6.** Supplemental Figure 7**. Sensitivity analyses: multivariate path analysis model 7.** Supplemental Table 1**. Comparisons in characteristics between patients who survived to discharge and those who did not.

## Abbreviations

ACLS: Advanced cardiac life support
AEDs: Automated external defibrillators
CI: Confidence interval
CPR: Cardiopulmonary resuscitation
CVD: Cardiovascular disease
EMS: Emergency Medical Service
IQR: Interquartile range
OHCA: Out-of-hospital cardiac arrest
ROSC: Return of spontaneous circulation

## Declarations

### Availability of data and materials

The datasets used or analysed during the current study are available from the corresponding author on reasonable request.
