# Supplementary material for: Trend in survival after out-of-hospital cardiac arrest and its relationship with bystander cardiopulmonary resuscitation: a six-year prospective observational study in Beijing
Source: BMC Cardiovasc Disord. 2021 Dec 31;21:625. doi: 10.1186/s12872-021-02446-z (PMC8720208; doi:10.1186/s12872-021-02446-z)
Supplement: Supplementary file 1 — Additional file 1. Supplemental Figure 1. The map of Emergency Medical Services centers in Beijing. Supplemental Figure 2. Survival rates of out-of-hospital cardiac arrest among bystanders with previous CPR training. Supplemental Figure 3. Sensitivity analyses: multivariate path analysis model 3. Supplemental Figure 4. Sensitivity analyses: multivariate path analysis model 4. Supplemental Figure 5. Sensitivity analyses: multivariate path analysis model 5. Supplemental Figure 6. Sensitivity analyses: multivariate path analysis model 6. Supplemental Figure 7. Sensitivity analyses: multivariate path analysis model 7. Supplemental Table 1. Comparisons in characteristics between patients who survived to discharge and those who did not. [file 12872_2021_2446_MOESM1_ESM.docx]

**Online Supplement**

**
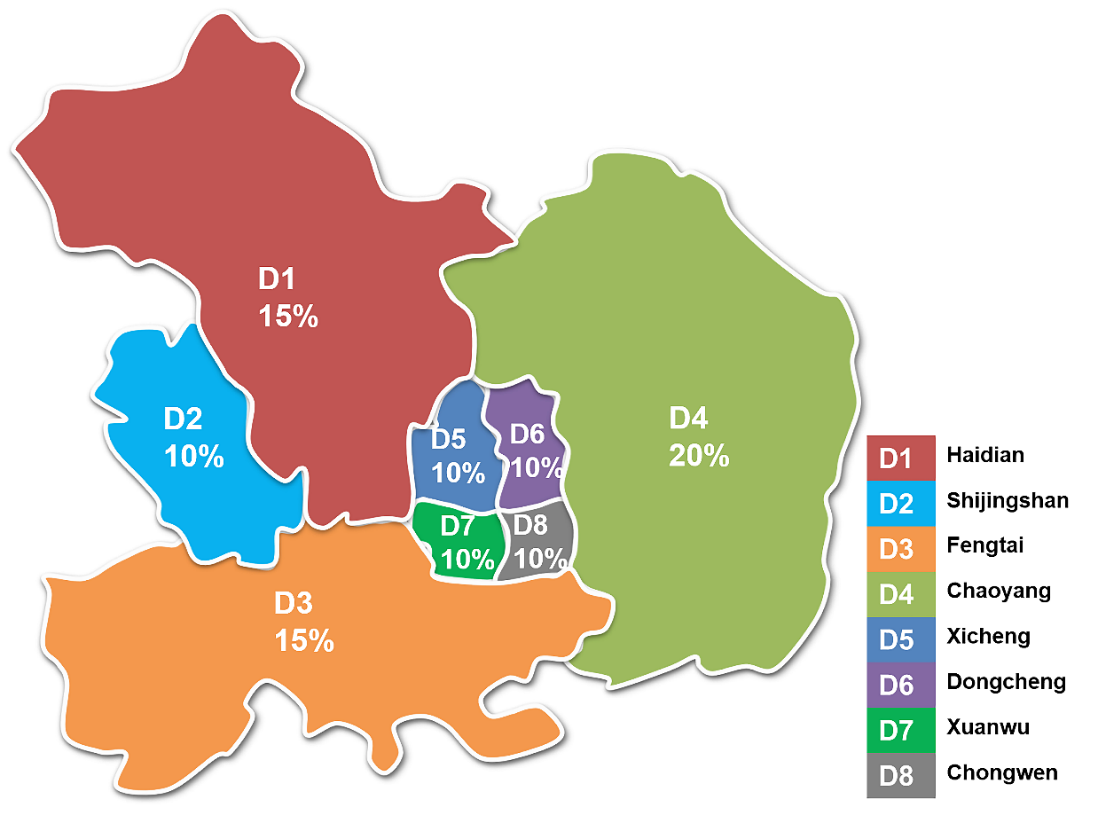
**

**Supplemental Figure 1. The map of Emergency Medical Services centers in Beijing.**

Cases of out-of-hospital cardiac arrest that occurred in the Beijing urban area (includes eight districts) and that were served by Beijing 120 center were included in this study. D, district.

**
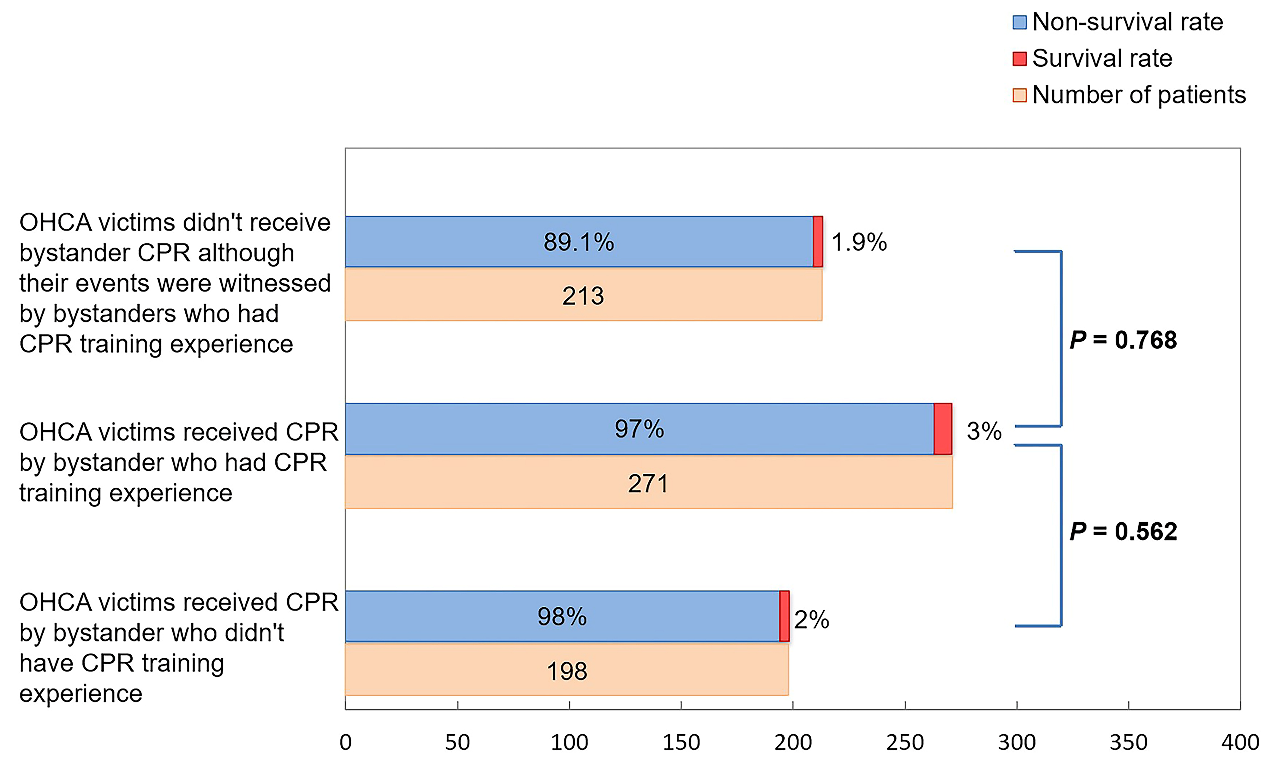
**

**Supplemental Figure 2. Survival rates of out-of-hospital cardiac arrest.**

OHCA, out-of-hospital cardiac arrest; CPR, cardiopulmonary resuscitation.


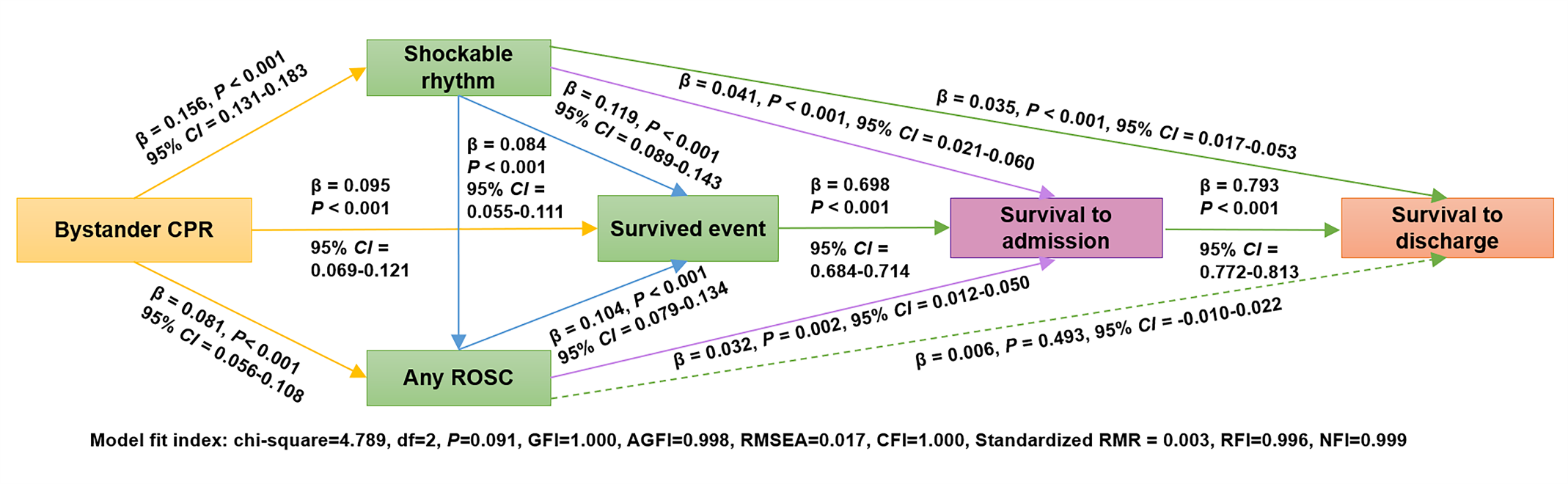


**Supplemental Figure 3. Multivariate path analysis model 3 (N = 5014, assuming that survival to admission was a mediator between survived event and survival to discharge)**

ROSC, return of spontaneous circulation; CPR, cardiopulmonary resuscitation; GFI, goodness-of-fit index; AGFI, adjusted goodness-of-fit index; RMSEA, root mean square error of approximation; CFI, comparative fit index; Standardized RMR, standardized root mean squared residual; RFI, relative fit index; NFI, normed fit index; CI, confidence interval; β, unadjusted estimate.


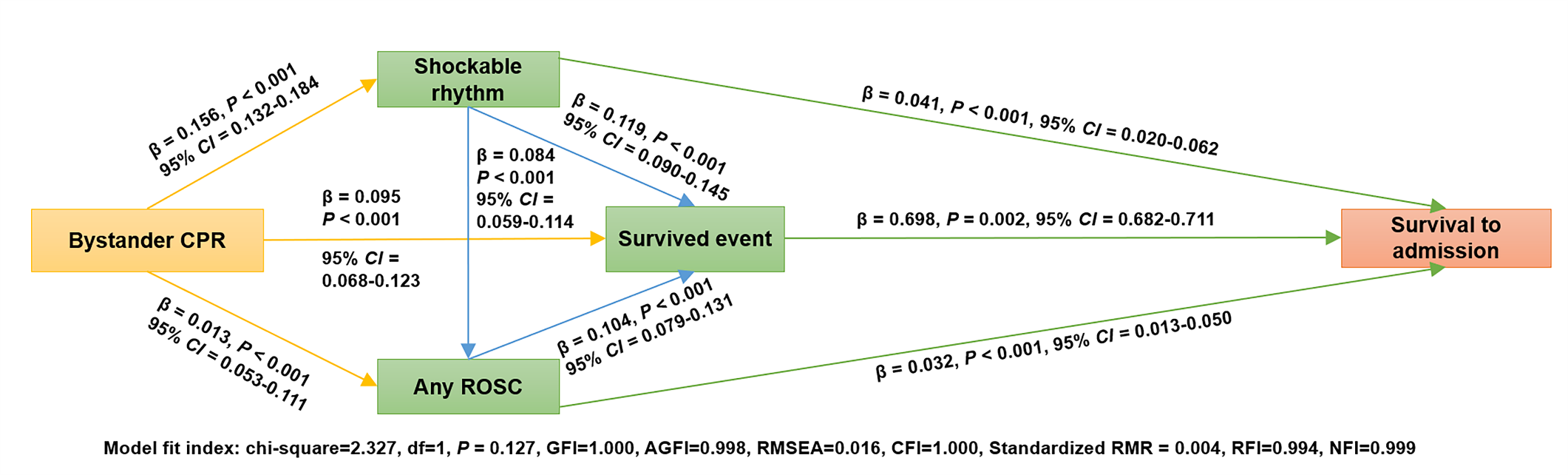


**Supplemental Figure 4. Multivariate path analysis model 4 (N = 5014, using survival to admission as a dependent variable)**

ROSC, return of spontaneous circulation; CPR, cardiopulmonary resuscitation; GFI, goodness-of-fit index; AGFI, adjusted goodness-of-fit index; RMSEA, root mean square error of approximation; CFI, comparative fit index; Standardized RMR, standardized root mean squared residual; RFI, relative fit index; NFI, normed fit index; CI, confidence interval; β, unadjusted estimate.

**
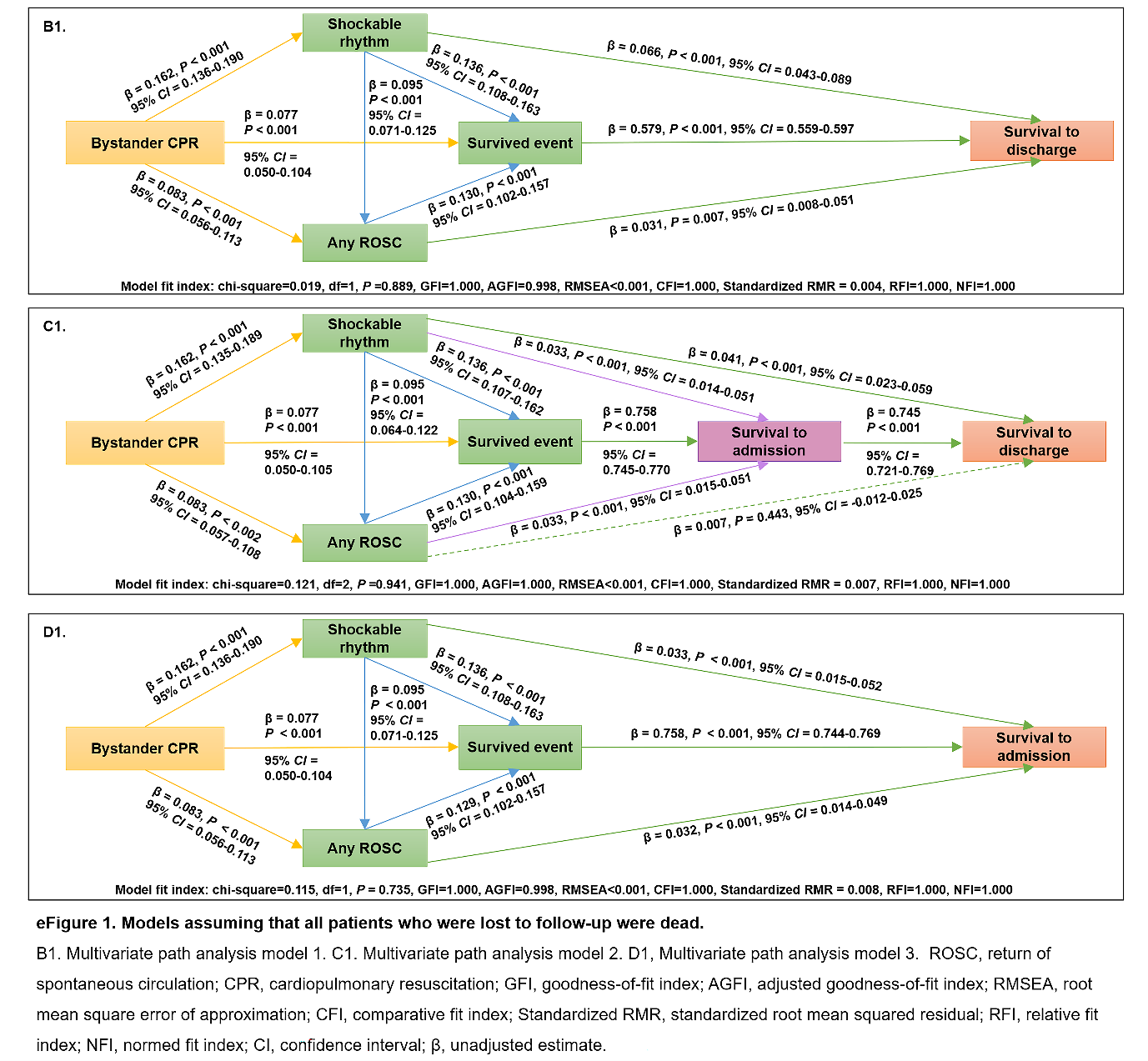
**

**Supplemental Figure 5. Multivariate path analysis model 5 (N = 5042, assuming that all patients who were lost-to-follow-up were dead)**

ROSC, return of spontaneous circulation; CPR, cardiopulmonary resuscitation; GFI, goodness-of-fit index; AGFI, adjusted goodness-of-fit index; RMSEA, root mean square error of approximation; CFI, comparative fit index; Standardized RMR, standardized root mean squared residual; RFI, relative fit index; NFI, normed fit index; CI, confidence interval; β, unadjusted estimate.

**
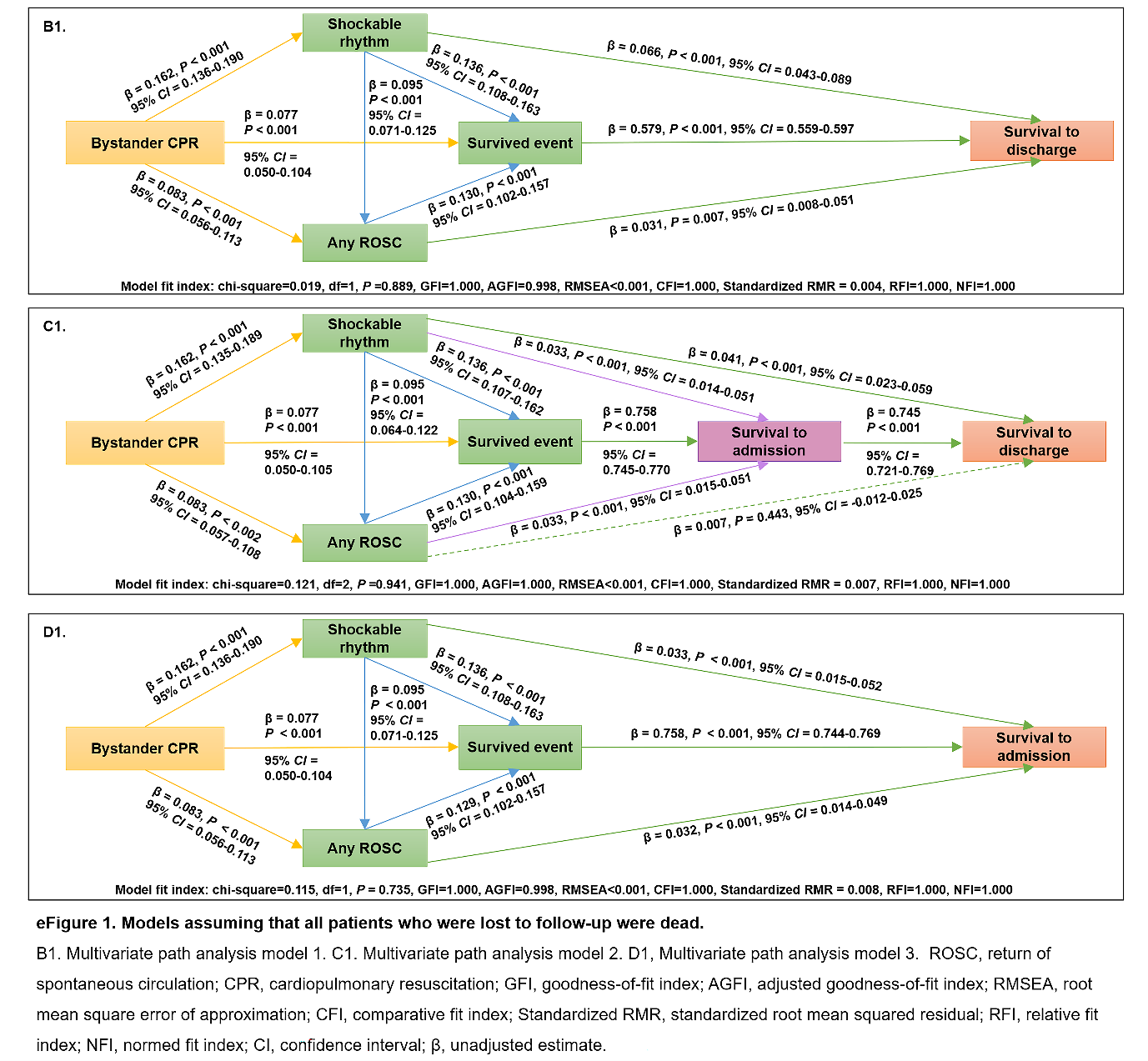
**

**Supplemental Figure 6. Multivariate path analysis model 6 (N = 5042, assuming that all patients who were lost-to-follow-up were dead and assuming that survival to admission was a mediator between survived event and survival to discharge)**

ROSC, return of spontaneous circulation; CPR, cardiopulmonary resuscitation; GFI, goodness-of-fit index; AGFI, adjusted goodness-of-fit index; RMSEA, root mean square error of approximation; CFI, comparative fit index; Standardized RMR, standardized root mean squared residual; RFI, relative fit index; NFI, normed fit index; CI, confidence interval; β, unadjusted estimate.

**
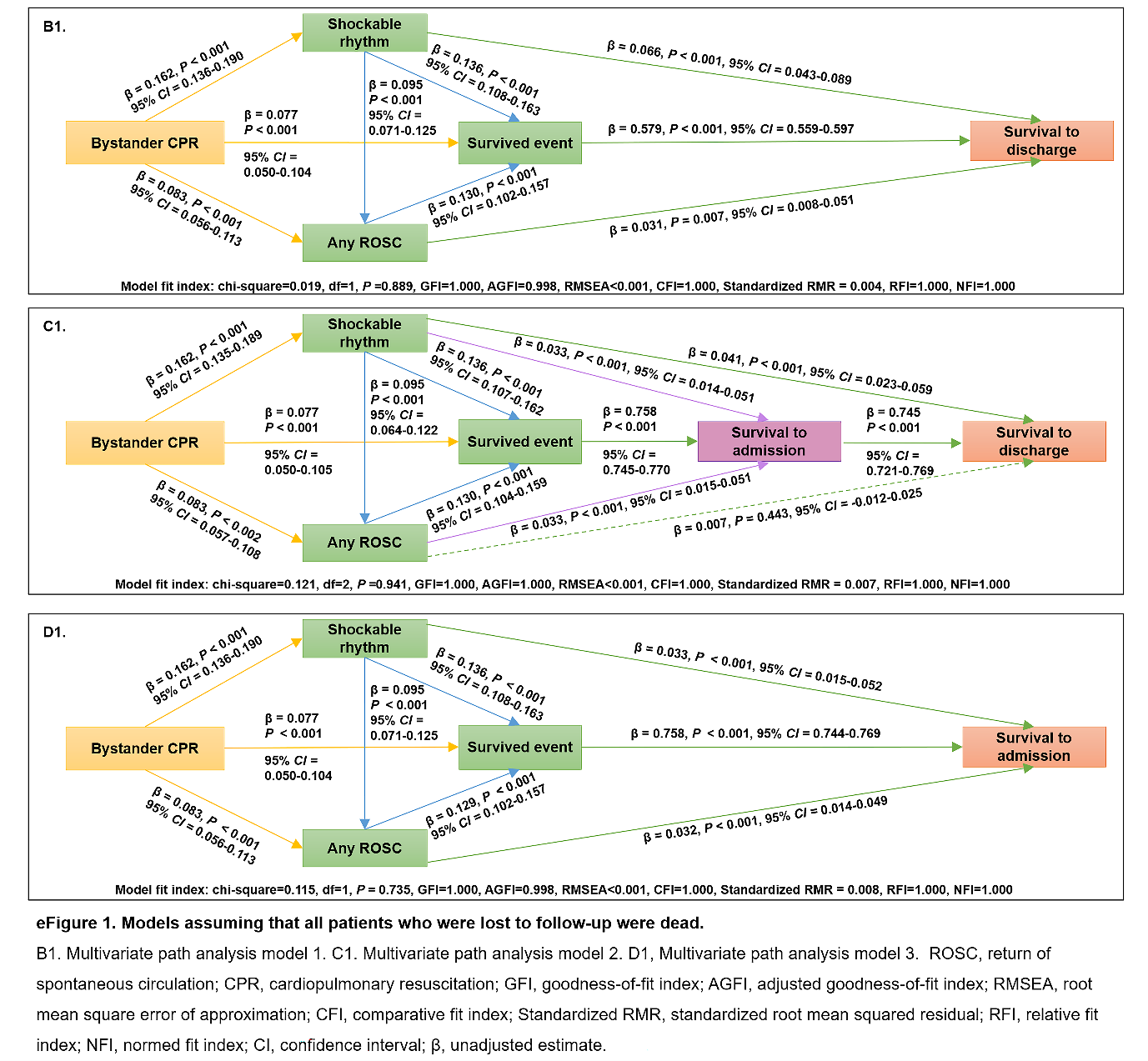
**

**Supplemental Figure 7. Multivariate path analysis model 7 (N = 5042, assuming that all patients who were lost-to-follow-up were dead and using survival to admission as a dependent variable)**

ROSC, return of spontaneous circulation; CPR, cardiopulmonary resuscitation; GFI, goodness-of-fit index; AGFI, adjusted goodness-of-fit index; RMSEA, root mean square error of approximation; CFI, comparative fit index; Standardized RMR, standardized root mean squared residual; RFI, relative fit index; NFI, normed fit index; CI, confidence interval; β, unadjusted estimate.

Supplemental Table 1. Comparisons in characteristics between patients who survived to discharge and those who did not

| Characteristics | Transported to hospital with ROSC (N = 95) ^&^ | | | Transported to hospital with ongoing bystander CPR (N = 389) ^&^ | | |
| --- | --- | --- | --- | --- | --- | --- |
|  | Survival to Discharge  (n = 30) | Non-survival  (n = 65) | *P* | Survival to Discharge  (n = 12) | Non-survival  (n = 377) | *P* |
| Age, median (IQR) | 64 (52-73) | 73 (56-80) | 0.030 | 61 (51-74) | 68 (57-80) | 0.119 |
| Gender |  |  | 0.315 |  |  | 0.090 |
| Male | 19 (63.3) | 34 (52.3) |  | 6 (50.0) | 273 (72.4) |  |
| Female | 11 (36.7) | 31 (47.7) |  | 6 (50.0) | 104 (27.6) |  |
| Arrest location |  |  | 0.018 |  |  | 0.027 |
| At home | 17 (56.7) | 52 (80.0) |  | 6 (50.0) | 292 (77.5) |  |
| At public place | 13 (43.3) | 13 (20.0) |  | 6 (50.0) | 85 (22.5) |  |
| Witnessed status |  |  | 0.892 |  |  | 0.114 |
| Non-witnessed | 7 (23.3) | 16 (24.6) |  | 1 (8.3) | 121 (32.1) |  |
| Witnessed | 23 (76.7) | 49 (75.4) |  | 11 (91.7) | 256 (67.9) |  |
| Bystander CPR |  |  | 0.904 |  |  | - |
| Yes | 11 (36.7) | 23 (35.4) |  | - | - |  |
| No | 19 (63.3) | 42 (64.6) |  | - | - |  |
| Bystander CPR training |  |  | 0.469 |  |  | 0.564 |
| Yes | 11 (36.7) | 19 (29.2) |  | 8 (66.7) | 212 (56.2) |  |
| No | 19 (63.3) | 46 (70.8) |  | 4 (33.3) | 165 (43.8) |  |
| First monitored shockable rhythm |  |  | 0.011 |  |  | 0.045 |
| Yes | 11 (36.7) | 9 (13.8) |  | 4 (33.3) | 43 (11.4) |  |
| No | 19 (63.3) | 56 (86.2) |  | 8 (66.7) | 334 (88.6) |  |
| Etiology |  |  | 1.000 |  |  | 1.000 |
| Cardiac | 25 (83.3) | 55 (84.6) |  | 11 (91.7) | 344 (91.2) |  |
| Non-cardiac | 5 (16.7) | 10 (15.4) |  | 1 (8.3) | 33 (8.8) |  |
| Previous CVD |  |  | 0.593 |  |  | 0.538 |
| Yes | 13 (43.3) | 32 (49.2) |  | 7 (58.3) | 252 (66.8) |  |
| No | 17 (56.7) | 33 (50.8) |  | 5 (41.7) | 125 (33.2) |  |
| Response time (min), median (IQR) | 15 (10-19) | 14 (11-17) | 0.593 | 12 (9-17) | 15 (12-19) | 0.082 |
| Any ROSC |  |  | 0.224 |  |  | 0.028 |
| Yes | 6 (20.0) | 7 (10.8) |  | 3 (25.0) | 20 (5.3) |  |
| No | 24 (80.0) | 58 (89.2) |  | 9 (75.0) | 356 (94.7) |  |
| Survived event |  |  | - |  |  | < 0.001 |
| Yes | 30 (100.0) | 65 (100.0) |  | 11 (91.7) | 23 (6.1) |  |
| No | 0 | 0 |  | 1 (8.3) | 354 (93.9) |  |
| Survived to admission |  |  | < 0.001 |  |  | < 0.001 |
| Yes | 30 (100.0) | 19 (29.2) |  | 12 (100.0) | 9 (2.4) |  |
| No | 0 | 46 (70.8) |  | 0 | 368 (97.6) |  |

Data are shown as frequency (percentage) or median (interquartile range). CPR, cardiopulmonary resuscitation; CVD, cardiovascular disease; IQR, interquartile range; ROSC, return of spontaneous circulation. ^&^ Excluding patients who were lost to follow-up.
